# B-cell epitope prediction for developing allergy vaccines and their companion immunodiagnostics

**DOI:** 10.3389/fimmu.2025.1624339

**Published:** 2025-07-02

**Authors:** Robbi Miguel G. Falcon, Serina U. Fahrenbach, Louise Claire E. Ortiz, Salvador Eugenio C. Caoili

**Affiliations:** Biomedical Innovations Research for Translational Health Science (BIRTHS) Laboratory, Department of Biochemistry and Molecular Biology, College of Medicine, Manila, Philippines

**Keywords:** allergy, vaccines, B-cell epitope prediction, B-cell epitopes, immunodominance, oligopeptide sequences, antipeptide antibodies, desensitization therapy

## 1 Introduction

Allergy is hypersensitivity (i.e., maladaptive proinflammatory immune reactivity) to noninfectious nonself antigens (i.e., allergens) such as environmental and food components, most notably in the context of immediate-type hypersensitivity, which is mediated by IgE antibodies (1). Binding of allergens by IgE antibodies thus underlies immunodiagnostic detection of allergy; and attenuation of such binding enables immunotherapeutic management of allergy. This is the rationale for desensitization therapy that entails detecting IgE antibodies to pertinent allergens among patients who are then immunized with the same allergens, thereby eliciting production of cognate non-IgE (typically IgG) antibodies to outcompete IgE antibodies in binding the allergens and thus alleviate allergic conditions (2). However, preexisting IgE antibodies can mediate harmful allergic reactions (e.g., fatal systemic anaphylaxis) when patients are exposed to cognate allergens, especially during desensitization therapy (3). Nevertheless, a translational path toward safer desensitization therapy for allergies is conceivable via development of allergy vaccines and their companion immunodiagnostics, using B-cell epitope prediction (BCEP) as a generally applicable strategy for enhancing disease control and prevention (4). When compared to allergen extract-based immunotherapies, B-cell epitopes (BCEs) have shown promising potential both *in vivo* and *in vitro*, by inducing hypoallergenic allergen-specific IgG responses and downregulating T-cell mediated late response pathways (5–9).

## 2 BCEP for designing allergy vaccines

BCEP is computational identification of BCEs: structural features (e.g., parts of molecules or of supramolecular complexes) recognized by paratopes (i.e., antigen-binding sites on immunoglobulins) (4). This is complicated by the emergent phenomenon of immunodominance among BCEs, which is the bias of antibody responses toward a BCE on an immunogen (i.e., immunogenic antigen) that comprises nonidentical BCEs. A BCE is thus said to be immunodominant if antibodies are preferentially produced against it rather than another BCE also present on the same immunogen (e.g., a vaccine antigen); and the latter BCE is thus said to be subdominant, though antibodies might yet be produced against it under other circumstances (e.g., where it is the sole or most immunodominant BCE present, as in a vaccine devoid of more immunodominant BCEs).

Among peptidic (e.g., peptide or protein) antigens, a typical BCE may be regarded as consisting of paratope-contacting amino-acid residues (10). Such a BCE is said to be either continuous if its constituent residues form a contiguous sequence or discontinuous otherwise (e.g., where the BCE is formed by juxtaposition of noncontiguous residues via protein folding and disintegrates upon protein unfolding), noting that a discontinuous BCE may comprise one or more continuous BCEs (11, 12). A continuous BCE is thus embodied in an oligopeptide sequence without regard to conformation; whereas a discontinuous BCE exists only when its antigen assumes a folded conformation. Consequently, BCEP is more computationally tractable for continuous BCEs than for discontinuous BCEs insofar as it reduces to identification of oligopeptide sequences. This is the case for peptide-based vaccine design, wherein the problem of immunodominance among BCEs can be circumvented by selectively incorporating only continuous BCEs into vaccine immunogens, to enable vaccine-induced selective antibody targeting of BCEs (13) via paratope binding that is typically based on induced fit (14).

To thus apply BCEP for designing allergy vaccines, the following general observation is key: Antibody responses to folded protein antigens (e.g., typical allergens) tend to be biased toward production of antibodies to discontinuous rather than continuous BCEs (10). Although this has been interpreted as implying that most protein BCEs are discontinuous, it is a clear manifestation of immunodominance among BCEs, with discontinuous BCEs tending to be more immunodominant than continuous BCEs that are nonetheless immunogenic (e.g., as unfolded parts of denatured proteins or as oligopeptides) (15). In the setting of allergy, most clinically relevant IgE antibodies thus recognize discontinuous BCEs (16). Furthermore, multiple BCEs recognized by IgE antibodies may occur on a single allergen (17), such that it can crosslink cognate IgE antibodies bound by FcϵRI receptors on plasma membranes of mast cells and of basophils (18, 19), thereby inducing degranulation with consequent extracellular release of inflammatory mediators (e.g., histamine) that drive allergic reactions (20). Yet, IgG antibodies can readily be produced against continuous BCEs that are conformationally disordered (i.e., nonfolded) oligopeptide sequences (e.g., in synthetic peptide-based vaccines); and if these sequences are also present as paratope-accessible targets in protein antigens (e.g., on surface-exposed conformationally flexible loops), they can be bound as such by the same antibodies (4). Hence, peptidic allergens can be targeted by IgG antibodies that recognize continuous BCEs, to competitively interfere with binding of the allergens by IgE antibodies (e.g., via steric blocking) and/or to enhance immunological clearance of the allergens (e.g., via IgG-dependent opsonization), noting that IgG antibodies other than IgG4 antibodies (21) can also drive shifts from allergy-promoting Th2-dominated to tolerogenic Treg-dominated immune responses (22). This must, however, still address possible cross-reactivity whereby nonidentical (albeit typically similar) BCEs can be bound by the same antibodies (23), which is a safety concern as existing IgE antibodies may thus cross-react with BCEs on non-cognate allergens (24).

Accordingly, oligopeptide sequences comprising continuous BCEs of protein allergens could conceivably serve as components of both allergy vaccines and corresponding companion immunodiagnostics: The vaccines could elicit production of IgG antibodies to the BCEs and thereby attenuate the allergy-mediating activity of IgE antibodies to the allergens while possibly also suppressing further production of IgE antibodies; whereas the immunodiagnostics could detect antibodies to the BCEs, to assess vaccine safety and efficacy. More specifically, certain versions of the immunodiagnostics could detect preexisting IgE antibodies to the BCEs before attempts to administer the vaccines (e.g., for primary and/or booster doses), so as to avoid triggering allergic reactions to the vaccines; whereas other versions of the immunodiagnostics could detect vaccine-induced IgG antibodies to the BCEs, in order to evaluate vaccine efficacy (noting that evidence of waning vaccine immunity might warrant subsequent booster doses). More generally, the vaccines could enable production of allergen-binding IgG antibodies (e.g., as monospecific polyclonal antibodies or even monoclonal antibodies) for possible therapeutic use via passive immunization (25), which would avoid risking vaccine-induced allergic reactions altogether (26) and could also serve as a preliminary trial of therapy that, if successful, might justify longer-term management by vaccination (i.e., active immunization).

**Figure 1:**
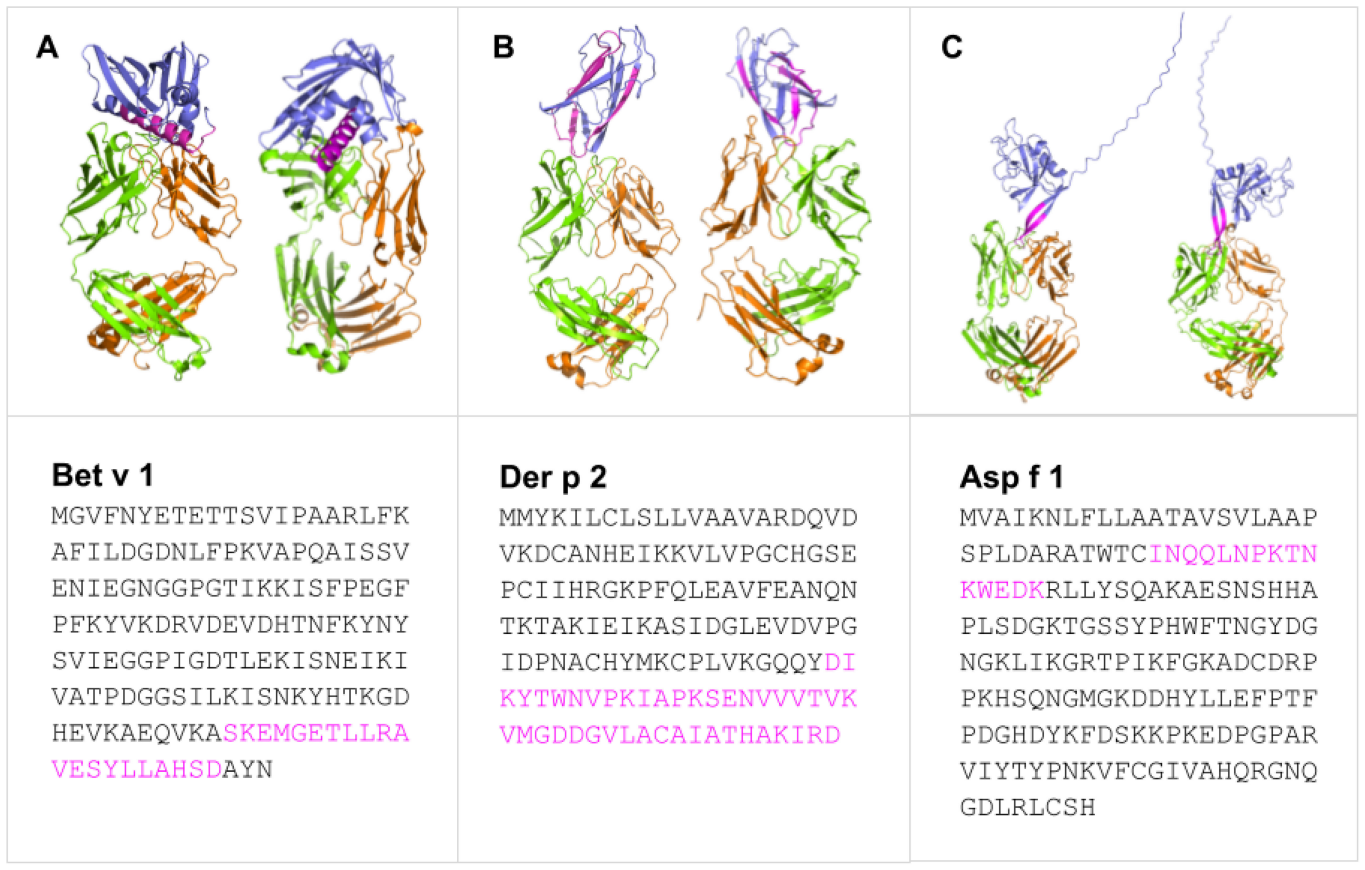
Predicted binding modes of Bet v 1 **(A)**, Der p 2 **(B)**, and Asp f 1 **(C)** (blue) epitopes (pink) with representative IgE and IgG Fab fragments showing heavy (green) and light (orange) chains. Generated with SWISS-MODEL Workspace (44) and PyMOL Molecular Graphics System ver 3.1.3.1.

In line with the preceding considerations, selective incorporation of continuous BCEs from allergens into components of allergy vaccines and companion immunodiagnostics thereto necessitates means for identifying such BCEs in the first place. This entails identification of relevant allergens and, in turn, their pertinent continuous BCEs. These can be validated only on the basis of experimental data from various immunoassays (27), notably as curated in the Immune Epitope Database (IEDB) (28). As a case in point, **Table 1** presents examples of IEDB-curated allergens and oligopeptide sequences thereof, for which active immunization with the latter is known to induce a decrease in allergic disease (noting that said sequences are each curated as a BCE in IEDB, though they are more properly regarded as BCE-containing sequences that each comprise one or more BCEs); whereas **Figure 1** presents structural models of a subset of the oligopeptide sequences in the context of immune complexes each consisting of a whole protein allergen and a cognate antibody Fab fragment, with surface-exposed BCE-containing sequences that comprise disorder-prone terminal or internal loop (e.g., turn) structures. Such examples illustrate the potential of oligopeptide sequences as allergy-vaccine components; but immunoassays are resource-intensive to perform and must be complemented by computational approaches including BCEP to facilitate identification of pertinent protein allergens and continuous BCEs thereof, using available biomolecular data on allergen structure and function in the context of host immunobiology to strategically target allergen BCEs.

**Table 1:** Allergen epitope sequences curated in the Immune Epitope Database (IEDB) as inducing decreased allergic disease.

#	Reference/s	Allergen (description)	Source organism	Classification	Epitope sequence
1	(29, 30)	Cry j 2 (Polygalacturonase precursor)	*Cryptomeria japonica*	Glycosyl hydrolase 28 family*; Pectin lyase-like superfamily protein 1*	AEVSYVHVNGAK
2	(31)	Der p 2 (Mite group 2 allergen Der p 2)	*Dermatophagoides pteronyssinus*	MD-2-related lipid-recognition (ML) domain; Group 2 mite allergen; NPC2 family*	DIKYTWNVPKIAPKSENVVVTVKVMGDDGVLACAIATHAKIRD
3	(32, 33)	Der p 1 (Der P 1)	*Dermatophagoides pteronyssinus*	Papain-like cysteine protease; Peptidase C1 family*	FGISNYCQIYPPNANKIREALAQPQRYCR
4	(34)	Asp f 1 (allergen I/a; Asp f I/a)	*Aspergillus fumigatus*	Ribonuclease U2 family*	INQQLNPK
5	(34)	Asp f 1 (allergen I/a; Asp f I/a)	*Aspergillus fumigatus*	Ribonuclease U2 family*	INQQLNPKTNKWEDK
6	(35)	Fel d 1 (Major allergen I polypeptide chain 1 precursor)	*Felis catus*	Secretoglobin family*	KALPVVLENARILKNCVDAKMTEEDKE
7	(34)	Asp f 1 (allergen I/a; Asp f I/a)	*Aspergillus fumigatus*	Ribonuclease U2 family*	LNPKTNKWEDK
8	(36)	Phl p 1 (Pollen allergen Phl p 1 precursor)	*Phleum pratense*	Group1/2/3 grass pollen allergen; Expansin family	LRSAGELELQFRRVKCKYPEG
9	(36–40)	Bet v 1 (major allergen Bet v 1)	*Betula pendula*	Pathogenesis-related (PR-10) protein; Bet v 1 family	MGETLLRAVESYLL
10	(36–40)	Bet v 1 (major allergen Bet v 1)	*Betula pendula*	Pathogenesis-related (PR-10) protein; Bet v 1 family	SKEMGETLLRAVESYLLAHSD
11	(32, 33)	Der p 1 (Der p 1 allergen precursor)	*Dermatophagoides pteronyssinus*	Papain-like cysteine protease; Peptidase C1 family*	SNYCQIYPPNANKIR
12	(41)	Major pollen allergen Ole e 1-like (Major pollen allergen)	*Olea europaea*	Ole e I family	TVNGTTRTVNPLGFFKKEALPK
13	(36)	Phl p 5 (major allergen Phl p 5)	*Phleum pratense*	Group 5/6 grass pollen allergen; Poa p IX/Phl p VI allergen family*	YAATVATAPEVKYTVFETALKKAI
14	(42)	Dol m 5 (Venom allergen 5.01)	*Dolichovespula maculata*	CRISP family; Venom allergen 5-like subfamily*	IEDNWYTHYLVCNYGPGGND
15	(42)	Api m 4 (melittin)	*Apis mellifera*	Melittin family*	KVLTTGLPALISW
16	(29, 30, 43)	Cry j 2	*Cryptomeria japonica*	Glycosyl hydrolase 28 family*; Pectin lyase-like superfamily protein 1*	CNFAAAGRFTCQTG
17	(31)	Der p 2	*Dermatophagoides pteronyssinus*	MD-2-related lipid-recognition (ML) domain; Group 2 mite allergen; NPC2 family*	TANINFECPRELVVPGGCN

*Based on sequence similarity, as curated in UniProt.

## 3 Strategically targeting allergen BCEs

BCEs represent a low-level structural and functional view of antigens, but higher-level views are necessary to comprehend the role of various antigens as allergens, in order to subsequently identify their pertinent BCEs as potential therapeutic targets (e.g., for binding by IgG antibodies). In this regard, a useful analytical framework is epidemiologic transition theory (45), which seeks to explain temporal shifts in patterns of morbidity and mortality exemplified by the modernization-driven dual trend of decline in infectious diseases and rise in chronic inflammatory conditions such as allergy and autoimmunity (46). Said trend can be understood largely in terms of biota alteration theory (47), which posits that generalized suppression of the host biota (i.e., microbiota plus other symbionts such as helminths) via lifestyle changes (e.g., adoption of excessive infection-control measures and proinflammatory diets) has promoted host immunological dysregulation (48). By this account, allergy results from dysregulated production of IgE antibodies, though these are thought to have evolved as mediators of host immunity against peptidic toxins (e.g., of pathogenic bacteria and venomous animals) that can be inactivated by proteases from mast cells (49, 50).

Hence, peptidic antigens may elicit production of IgE antibodies and thus act as allergens if they cause direct host injury (e.g., via cellular or tissue damage due to proteolytic or membrane-permeabilizing activities) (51) or are recognized as danger signals via innate immune sensing mechanisms such as Toll-like receptors (TLRs) (52), especially in the setting of prolonged host exposure (e.g., due to their structural stabilization by disulfide bonds and consequent resistance to proteolytic degradation) (53). Moreover, such allergens often can undergo oligomerization (51), which in turn can facilitate crosslinking of FcϵRI receptor-bound IgE antibodies and consequent mast-cell degranulation (54). Immunotherapy and vaccine administration have previously been shown to predictably increase total serum IgE concentration and sIgE (55, 56) which may erroneously suggest a diagnosis of atopy. Thus, reinforcing the potential of BCEP as a predictive tool to minimize false-positive detection of sensitization among atopic individuals is necessary, given its capacity to streamline the development of BCEs for companion immunodiagnostic use (57).

In addition to host-damaging and danger-signaling activities vis-à-vis structural stability and oligomerization potential, allergens may be further characterized by more detailed structural features. Although allergens are structurally diverse, 19 allergen families have been identified from the Pfam database based on structural properties, with approaches to subclassification being explored mainly on the basis of homology. However, many allergens remain unclassified; and the structural properties underlying allergenicity are not yet fully understood, thus limiting BCE potential for widespread clinical use (52). Route of host exposure to antigens is also a crucial determinant of their clinical relevance as allergens (58): Food allergens, for instance, tend to enter the systemic circulation via transcytosis across the host gut lining epithelium and thus elicit production of IgE antibodies (59, 60). Such factors underlie the failure to develop experimental models that fully capture the complexity of allergic conditions (61, 62). This calls for computational workflows that can leverage data on protein sequences and structures vis-à-vis protein and immune-system function to aid in identifying clinically relevant protein allergens and/or BCEs thereof for therapeutic antibody targeting.

As thermodynamics provides a foundational framework for comprehending immune function (63), it could guide the use of computational tools (e.g., to predict protein folding and interactions) for analyzing pertinent proteomes (e.g., of cells or tissues in allergenic materials) to identify putative allergens (e.g., on the basis of predicted toxicity in particular contexts of relevant host exposure) and, in turn, candidate allergy vaccine BCEs via BCEP. Such BCEs could thus be identified as oligopeptide sequences for which sufficiently high paratope binding affinity is predicted (13), noting that paratopes that bind one BCE may fail to bind another even if the two are highly similar in sequence (64). Where identical BCEs occur on the same antigen, avidity (i.e., strength of binding due to simultaneous paratope-BCE interactions) might also be predicted (65). In this regard, steric hindrance is important to consider as it can attenuate avidity (66, 67), though it can also attenuate allergen toxicity as by inhibiting host-damaging protease activity via blockage of substrate access to active sites (68). Finally, the selected vaccine BCEs would also serve as companion immunodiagnostic probes to detect cognate antibodies, which would obviate the need for large peptide antigen arrays representing entire protein allergen sequences (69).

## 4 Conclusion

BCEP-based design of allergy vaccines and their companion immunodiagnostics using thermodynamics-guided computational workflows is a promising approach to further develop desensitization therapy for allergic conditions. This could enable strategic IgG-antibody targeting of key continuous BCEs, most notably to limit the binding of allergens by IgE antibodies while also avoiding potentially harmful exposure of patients to immunodominant allergen BCEs
